# Diversification and colonization processes in Gobioidei predicted based on mitochondrial 12S rRNA with focusing on Oxudercidae

**DOI:** 10.1080/23802359.2021.1901620

**Published:** 2021-03-26

**Authors:** Hyung-Bae Jeon, Jumin Jun, Seung-Ho Choi, Ho Young Suk

**Affiliations:** a Department of Biology, Concordia University, Montreal, Canada; b National Institute of Biological Resources, Environmental Research Complex, Incheon, Republic of Korea; c SOKN Institute of Ecology and Conservation, Seoul, Republic of Korea; d Department of Life Sciences, Yeungnam University, Gyeongsan, Republic of Korea

**Keywords:** Gobioidei, Gobiidae, Oxudercidae, 12S rRNA, phylogeny

## Abstract

Gobioidei is one of the largest vertebrate taxa with over 2000 species observed around the globe. The largest group in Gobioidei is gobies that had been classified as one family, Gobiidae, based on morphological features. Molecular phylogenetic studies revealed that gobies consisted of two monophyletic families, Gobiidae and Oxudercidae, in which 19 lineages have been proposed, despite some claims arisen about the relationship among these lineages or species. We analyzed 58 Gobioidei species, including 45 East Asian oxudercids, based on 12S rRNA sequences to reconstruct the spatiotemporal diversification history of gobies. Our analysis yielded the results compatible with the previous reports in a large framework. The common ancestor of Gobiidae and Oxudercidae were estimated to appear at 38.66 Mya. Genus-level splits occurred in Gobiidae and Oxudercidae predominantly at Miocene and late Miocene to early Pleistocene, respectively. Gobies have likely originated in many parts of the northern and western Pacific Ocean, of which a large proportion of Oxudercidae have adapted to various environments in the North Pacific.

## Introduction

Gobioidei (Gobiiformes) is one of the largest vertebrate taxa with over 2000 species (Nelson 2006). Gobioidei species of this suborder appear in all types of aquatic environments around the globe except Arctic and Antarctic regions (Nelson 2006), and is currently classified into nine families based on the morphological and molecular traits (Thacker 2009; Agorreta et al. 2013; Reichenbacher et al. 2020). The largest group in Gobioidei is gobies that had been classified as Gobiidae until recently (Nelson 2006; Nelson et al. 2016). As molecular tools were applied, there have been claims that Gobiidae could be divided into two monophyletic families, Gobiidae and Oxudercidae (Thacker 2003; Agorreta et al. 2013; Nelson et al. 2016). Recent studies further assigned these two families into 19 lineages, five from Oxudercidae and 14 from Gobiidae (Thacker and Roje 2011; Thacker 2013; Agorreta et al. 2013; McCraney et al. 2020). However, many Oxudercidae species were not considered in establishing this lineage system.

The majority of Gobiidae species inhabit intertidal and near-shore marine environments. However, oxudercid species appear in a variety of habitats, intertidal zones, brackish waters and freshwater (Patzner et al. 2011), suggesting the possibility that adaptive radiation was as an important process in the speciation (Thacker 2009). Given the biogeographic assumption that gobies originated in the Western Indian Ocean and West Tethys (Dornburg et al. 2014; Thacker 2015), it is conceivable that huge expansions of oxudercid distribution have been made to shape the contemporary distributions, during which the speciation may have occurred by colonizing new regions (Agorreta et al. 2013; Thacker 2015).

Our goal was to reexamine the spatiotemporal diversification history of major lineages in Gobioidei with analyzing new Oxudercidae species. We compiled mitochondrial 12S rRNA sequences from 134 Gobioidei species, of which 58 (including 45 East Asian oxudercids) were sequenced and 76 were retrieved from GenBank (Table 1), to estimate the age and pattern of divergence. Although it is not easy to reconstruct a complete history only with a single gene region, 12S rRNA may be the best region allowing direct comparisons with previous Gobioidei studies. The ancestral distributions and habitat states were also predicted using our inferred phylogenetic frame.

**Table 1.** The list of species analyzed in this study and the sampling information.

	Family	Species	GPS coordinate	Date	Accn #
1	Odontobutidae	*Micropercops swinhonis*	N35°5′57″, E126°45′7″	10 May 2011	KM030423
2		*Odontobutis interrupta*	N36°2′24″, E129°14′41″	7 Dec 2010	KM030424
3		*Odontobutis obscura*	N34°48′51″. E128°38′8″	20 Jun 2011	KM030425
4		*Odontobutis platycephala*	N35°51′23″, E128°43′41″	23 Dec 2010	KM030426
5	Gobiidae	*Eviota abax*	N33°28′23″, E126°20′58″	21 Feb 2011	KM030449
6		*Istigobius campbelli*	N33°16′15″, E126°35′53″	9 Jun 2011	KM030447
7		*Istigobius hoshinonis*	N33°16′15″, E126°35′53″	9 Jun 2011	KM030446
8		*Bathygobius fuscus*	N33°28′23″, E126°20′58″	21 Feb 2011	KM030438
9		*Leucopsarion petersii*	N35°19′32″, E129°15′18″	4 Apr 2011	KM030448
10		*Favonigobius gymnauchen*	N33°28′23″, E126°20′58″	21 Feb 2011	KM030433
11		*Cryptocentrus fillifer*	N36°9′27″, E126°30′1″	9 Jun 2011	KM030439
12		*Parioglossus dotui*	N33°28′23″, E126°20′58″	21 Feb 2011	KM030436
13		*Asterropteryx semipunctata*	N33°16′16″, E126°39′34″	16 Nov 2011	KM030437
14	Oxudercidae	*Lophiogobius ocellicauda*	N37°42′7″, E126°39′45″	13 Oct 2011	KM030427
15		*Synechogobius hasta*	N36°21′6″, E126°33′36″	14 May 2011	KM030428
16		*Acanthogobius elongata*	N36°21′6″, E126°33′36″	15 May 2011	KM030429
17		*Acanthogobius flavimanus*	N36°21′6″, E126°33′36″	15 May 2011	KM030430
18		*Acanthogobius lactipes*	N36°21′6″, E126°33′36″	15 May 2011	KM030431
19		*Acanthogobius luridus*	N37°42′7″, E126°39′45″	13 Oct 2011	KM030432
20		*Amblychaeturichthys hexanema*	N36°9′27″, E126°30′1″	9 Jun 2011	KM030435
21		*Gymnogobius breunigii*	N38°20′8″, E128°30′57″	20 Aug 2011	KM030451
22		*Gymnogobius mororanus*	N36°21′6″, E126°33′36″	20 May 2011	KM030452
23		*Gymnogobius urotaenia*	N36°8′42″, E129°23′19″	19 Apr 2011	KM030455
24		*Gymnogobius petschiliensis*	N33°14′44″, E126°25′7″	25 Aug 2011	KM030454
25		*Gymnogobius opperiens*	N36°8′42″, E129°23′19″	19 Apr 2011	KM030453
26		*Chaeturichthys stigmatias*	N36°21′6″, E126°33′36″	14 May 2011	KM030450
27		*Chaenogobius annularis*	N33°28′23″, E126°20′58″	21 Feb 2011	KM030456
28		*Chaenogobius gulosus*	N33°28′23″, E126°20′58″	21 Feb 2011	KM030457
29		*Clariger cosmurus*	N35°17′30″, E129°15′37″	4 Apr 2011	KM030458
30		*Luciogobius grandis*	N35°17′30″, E129°15′37″	4 Apr 2011	KM030460
31		*Luciogobius guttatus*	N33°28′23″, E126°20′58″	21 Feb 2011	KM030461
32		*Luciogobius platycephalus*	N35°17′30″, E129°15′37″	4 Apr 2011	KM030463
33		*Luciogobius pallidus*	N33°17′30″, E126°22′28″	9 Jun 2011	KM030462
34		*Luciogobius elongatus*	N35°17′30″, E129°15′37″	4 Apr 2011	KM030459
35		*Inu saikaiensis*	N35°17′30″, E129°15′37″	4 Apr 2011	KM030464
36		*Mugilogobius abei*	N36°21′6″, E126°33′36″	14 May 2011	KM030465
37		*Mugilogobius fontinalis*	N33°28′23″, E126°20′58″	23 Aug 2011	KM030466
38		*Pseudogobius masago*	N36°21′6″, E126°33′36″	14 May 2011	KM030467
39		*Pterogobius elapoides*	N34°3′10″, E125°7′25″	13 Jul 2011	KM030468
40		*Pterogobius zacalles*	N34°3′10″, E125°7′25″	13 Jul 2011	KM030469
41		*Pterogobius zonoleucus*	N35°15′51″, E129°14′7″	5 Apr 2011	KM030470
42		*Rhinogobius brunneus*	N36°7′0″, E129°18′54″	15 Mar 2011	KM030471
43		*Rhinogobius nagoyae*	N34°26′40″. E127°30′45″	4 Jun 2011	KM030472
44		*Rhinogobius mizunoi*	N33°14′38″, E126°20′30″	6 Jul 2011	KM030473
45		*Rhinogobius similis*	N33°14′44″, E126°25′7″	23 Aug 2011	KM030474
46		*Rhinogobius giurinus*	N35°51′15″, E127°10′19″	7 Jul 2011	KM030475
47		*Tridentiger barbatus*	N37°38′15″, E126°32′20″	8 Nov 2011	KM030476
48		*Tridentiger brevispinis*	N36°21′6″, E126°33′36″	14 May 2011	KM030477
49		*Tridentiger bifasciatus*	N36°21′6″, E126°33′36″	14 May 2011	KM030480
50		*Tridentiger nudicervicus*	N36°21′6″, E126°33′36″	14 May 2011	KM030478
51		*Tridentiger obscurus*	N34°48′35″, E128°14′15″	28 Oct 2011	KM030479
52		*Tridentiger trigonocephalus*	N38°28′49″, E128°26′11″	19 Aug 2011	KM030481
53		*Boleophthalmus pectinirostris*	N34°47′31″, E127°23′46″	15 Aug 2011	KM030440
54		*Periophthalmus magnuspinnatus*	N36°21′6″, E126°33′36″	25 Aug 2011	KM030445
55		*Periophthalmus modestus*	N36°32′18″, E126°28′15″	9 Oct 2011	KM030444
56		*Taenoides* sp.	N37°42′7″, E126°39′45″	13 Oct 2011	KM030443
57		*Ctenotrypauchen microcephalus*	N35°25′44″, E126°25′41″	5 Aug 2011	KM030441
58		*Odontamblyopus rubicundus*	N35°25′44″, E126°25′41″	5 Aug 2011	KM030442

## Materials and methods

**Figure 1.**
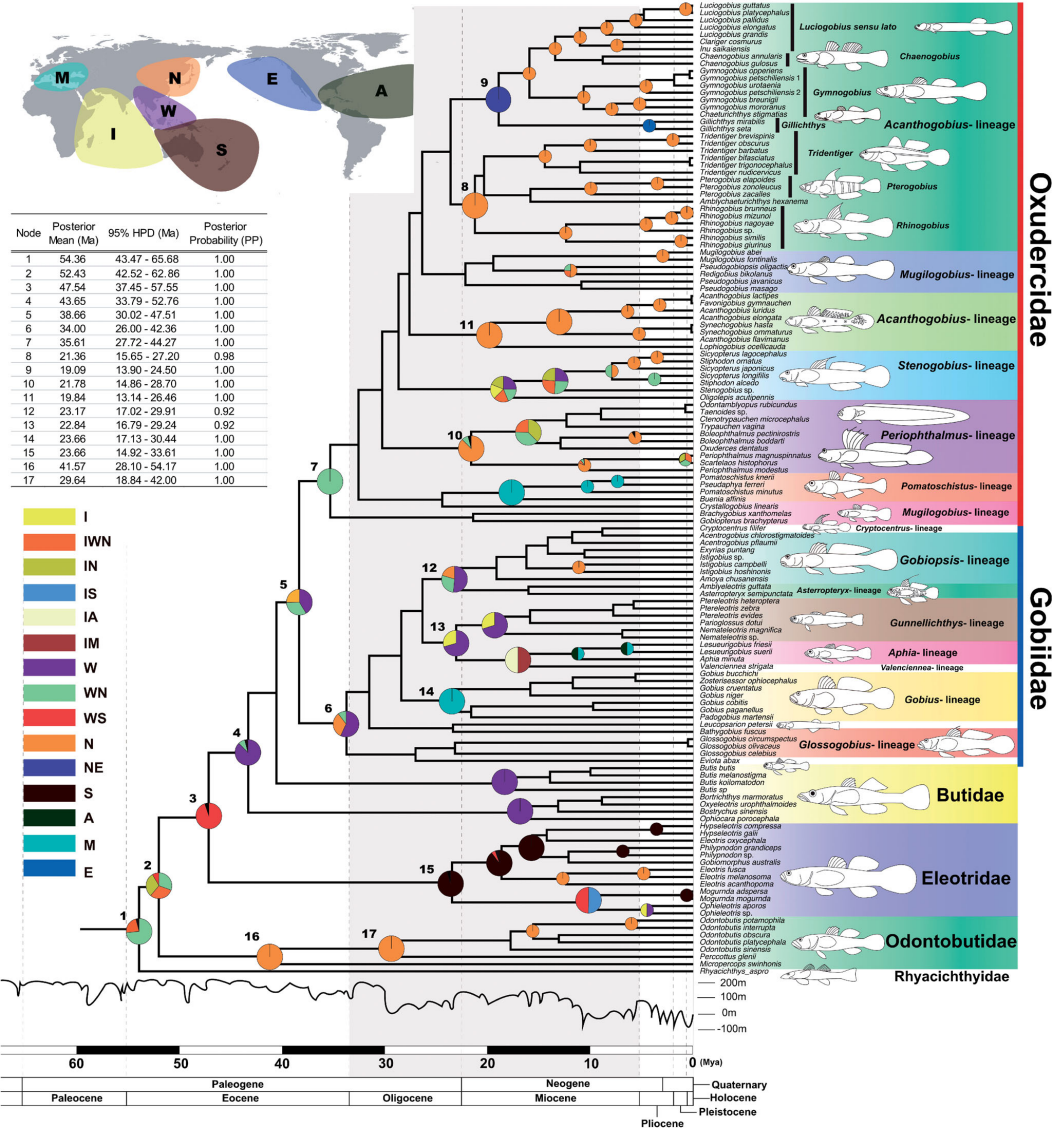
Time-calibrated Bayesian tree reconstructed by BEAST using 12S rRNA sequences of Gobioidei species (see for the comparison with BI tree, Supplementary Materials Figure S1). The biogeographic history of major clades was inferred under the statistical dispersal-vicariance analysis (S-DIVA) model of geographic range evolution using RASP on the frame of BEAST tree. The S-DIVA was performed by coding each species as occurring in one of the seven oceanographic regions which were defined on the basis of current distribution patterns (Supplementary Materials Table S1), (A) Atlantic Ocean, (E) East Pacific Ocean, (I) Indian Ocean, (M) Mediterranean Sea, (N) Northwest Pacific Ocean, (S) South Pacific Ocean and (W) West Pacific Ocean. The most likely ancestral areas were represented by the colors on the nodes that were generated by the combination of the seven oceanographic regions shown on the map.

12S rRNA was analyzed using 58 Gobioidei species stored as ethanol specimens at Yeungnam University that were collected from South Korea (Figure 1; Jeon et al. 2012). DNA was extracted from the caudal peduncles using the Wizard Genomic DNA purification kit (Promega). The targeted region was amplified using previously reported primers: 12S19F 5′-AAGCATAACACTGAAGATGTTAAG-3′ and 12S1063B 5′-CTCGGTGTAAGGGAGATG-3′ (Won et al. 2020); 16SA 5′-CGCCTGTTTAHCAAAAACAT-3′ and 16SB 5′-CCGCTYTGAACTCARATCA-3′ (Frankham 2005). PCR was performed with 50 μL of a volume composed of DNA extract, 0.25 mM of each deoxynucleotide, 0.25 mM of each primer, 3 mM MgCl_2_, 1X PCR buffer and 0.25 units of *Taq* polymerase (Solgent). GenePro (BIOER) were used for the amplification under the following profile: 94 °C for 5 m, 35 cycles of 30 s at 94 °C, 30 s at 52 °C (12S19F–12S1063B) or 60 °C (16SA–16SB), 30 s at 72 °C and final elongation at 72 °C for 10 m. PCR products were commercially sequenced at Genotech Inc. All novel sequences obtained were deposited at NCBI GenBank (Table 1). The DNA sequences were assembled and aligned using the GENEIOUS V5.4 (Kearse et al. 2012). The tRNA regions were excluded, and the secondary structure of 12 s rRNA was predicted following the procedures used in previous studies (see Wang et al. 2001).

JModelTest V2 (Darriba et al. 2012) was used to determine the best-fitting substitution model (GTR + G). Bayesian Inference (BI) analysis was conducted using MrBayes V3.2 (Ronquist et al. 2012) with independent MCMC analyses of 5,000,000 generations. Trees were sampled every 100 generations, and the first 25% generations were discarded as the burn-in. The 50% majority-rule consensus trees were created to estimate the posterior probability for recovered nodes. Parameters were checked for convergence using Tracer V1.6 (Rambaut et al. 2014). *Rhyacichthys aspro* was used as outgroup. Although this species belongs to Gobioidei, it makes sense to use this species, given that the target of our phylogenetic analysis was to gobies.

BEAST V1.6.1 (Drummond and Rambaut 2007) was used to estimate the divergence times among clades based on the BI tree reconstructed. The clock model was set to uncorrelated lognormal relaxed clock under a lognormal prior using the ‘Yule speciation process’, and the prior on the mean substitution rate was fixed to a lognormal distribution (mean ± SD = 0.002 ± 0.3). The point at which the branching of the gobioid started was set to Late Ypresian (47.8–56 MYA; Bannikov and Carnevale 2016). The MCMC was run to 10,000,000 generations, sampling the chain every 1000th step. MCMC convergence was estimated from the likelihood trace using Tracer. Each run was replicated three times independently, which were combined using LogCombiner V1.6.1 (Drummond and Rambaut 2007).

The biogeographic history of Gobioidei species was explored under the statistical dispersal-vicariance analysis (S-DIVA) model for ancestral state using RASP (Yu et al. 2015). Based on the BEAST result, JC model was applied under default setting. The analysis was performed by coding each species as occurring in one of the seven oceanographic regions (Froese and Pauly 2000; Figure 1). Ancestral habitat types were determined using Mesquite V2.75 (Maddison and Maddison 2011) based on the frame of BEAST tree, and the habitat information of the extant species (Froese and Pauly 2000).

## Results

A total of 575 sites were variable and parsimoniously informative in 12S rRNA sequences. All the analyzed families were well-resolved forming monophyletic groups with one exception (Butidae; Figure 1). Within the Oxudercidae, most genera and subfamilies formed clusters (lineages) in the pattern supporting previous reports (but see *Mugilogobius*-lineage in Figure 1). All genera of *Acanthogobius*-lineage formed a monophyletic group and were divided into two groups (node 8 and 9). *Periophthalmus*- and *Stenogobius*-lineage were the sisters of the *Acanthogobius*-lineage, and *Mugilogobius*-, *Pomatoschistus*- and *Acanthogobius*-lineage (node 11) diverged at the base in Oxudercidae. Gobiidae appeared as a monophyletic sister group to Oxudercidae. *Glossogobius*-lineage occupied the basal position in Gobiidae but not formed a monophyletic group being clustered with *Eviota abax* (Figure 1). The remaining gobiids were divided into three groups (node 12, 13 and 14). The sister taxon to the Oxudercidae and Gobiidae was *Butis* (Butidae; Figure 1). Eleotrids were the sister to Butidae, Oxudercidae and Gobiidae (Figure 1). Odontobutidae was located at the basal (Figure 1).

The root node for Gobioidei was estimated to be 54.36 Mya (Figure 1). Age estimates of 47.54 and 43.65 Mya were allocated to the nodes representing the separation of Eleotridae and Butidae, respectively (Figure 1). The most recent common ancestors (MRCA) of Oxudercidae and Gobiidae were estimated to appear at 38.66 (Figure 1). Within Oxudercidae and Gobiidae, the major lineages appeared around late Oligocene to late Miocene. Genus-level splits occurred predominantly from early to late Miocene in Gobiidae, whereas mid-Miocene to early Pleistocene in Oxudercidae (Figure 1).

**Figure 2.**
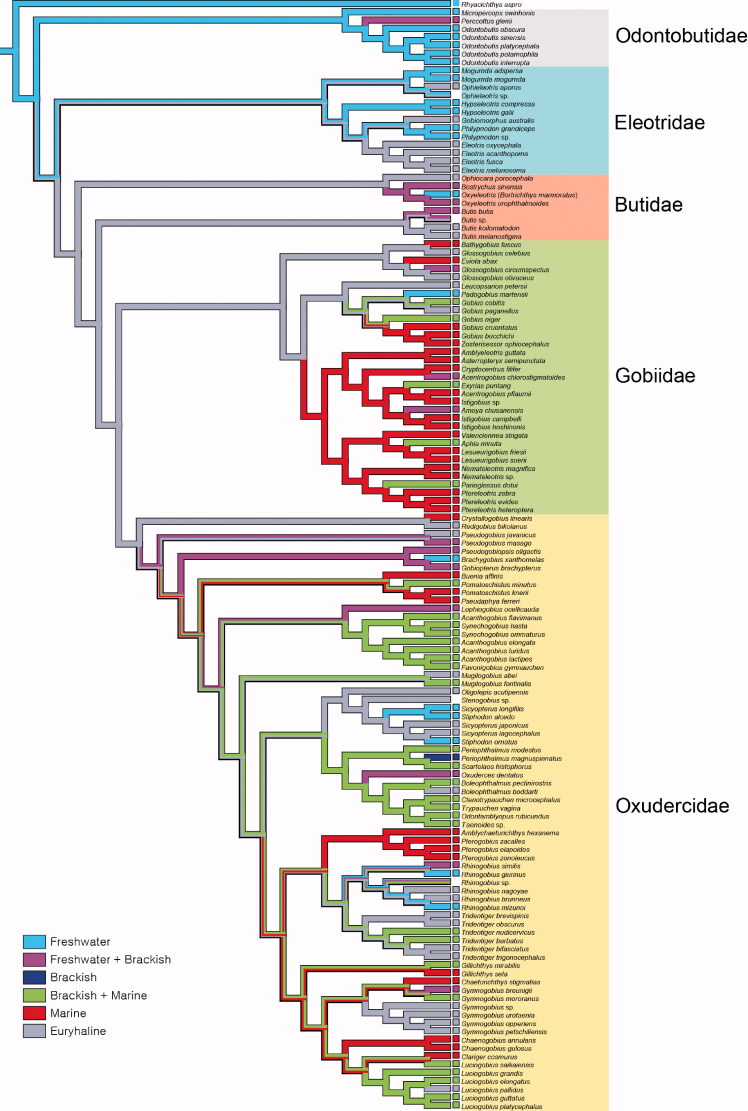
Ancestral habitat types reconstructed using Mesquite on the frame of BEAST tree. The habitat states of the extant species were defined as freshwater, brackish and marine (see Supplementary Materials Table S1 for details).

Our RASP result indicated that the MRCA of Gobioidei originated in the Indian Ocean, West and Northwest Pacific regions (node 2), and subsequently expanded its range (Figure 1). Odontobutidae likely diverged with occupying Northwest Pacific region (node 16), and the remaining taxa spread to West and South Pacific (node 3). Eleotridae was located and differentiated in South Pacific (node 15) and the remaining taxa were located in West Pacific (B; node 4). Butidae remained in the West Pacific, and Gobidae and Oxudercidae likely spread toward various regions (node 5). Oxudercidae likely originated from West and Northwest Pacific (node 7), whereas Gobiidae from various regions in the Pacific Ocean (node 6). Oxudercidae predominately located in the Northwest Pacific, though a few might migrate to Europe (*Pomatoschistus*-lineage) or North America (genus *Gillichthys*). The taxa located at the base in our phylogenetic tree were mostly freshwater species (Odontobutidae, Butidae and *Rhyacichthys aspro*; Figure 2), indicating that Gobioidei originated from freshwater environment. Gobiidae seems to predominantly have adapted to the marine and brackish habitats, whereas oxudercids appear to have diverged while adapting to a wider variety of environments (Figure 2). Within the Oxudercidae, there have been at least five times of habitat transition from euryhaline to marine (Figure 2).

## Discussion

In our results, a marked phylogenetic divergence was shown between Gobiidae and Oxudercidae, supporting the classification proposed by previous studies (see Introduction). *Butis* was placed as a sister group to the clade of Gobiidae and Oxudercidae (see also Thacker 2009). All *Glossogobius*-lineage species appeared as a monophyletic group and a sister to the remaining gobiids, though only two genera were analyzed in this study (see also Thacker 2009). *Glossogobius* species were divided into two clusters, *G. celebius* being grouped with *Bathygobius* and the rest with *Eviota abax* (*Gobiodon*-lineage). *Gunnellichthys*-lineage was resolved to form a complete monophyletic group. *Aphia*-lineage showed a sister relationship with *Valenciennea*-lineage (see also Thacker 2015; McCraney et al. 2020). Although *Gobiopsis*-lineage was resolved as a monophyletic group in previous reports (Thacker 2009; Agorreta et al. 2013), *Cryptocentrus*-lineage was clustered with *Gobiopsis*-lineage in our results.

Our analyses revealed that Oxudercidae could be divided into five sub-clades (see also Thacker 2015; McCraney et al. 2020), though the *Acanthogobius*-lineage failed to form a monophyletic clustering. *Rhinogobius* and *Stenogobius* were phylogenetically close with each other in previous studies (Parenti and Thomas 1998; Wang et al. 2001), which is clearly contrast to our results. *Periophthalmus*-lineage appeared as a sister group to *Stenogobius*-lineage (see also Agorreta et al. 2013; Thacker 2015). Although there have been claims to exclude Amblyopinae from Oxudercidae (Murdy and Shibukawa 2002), the common conclusion of recent studies is to maintain it within this family (Agorreta et al. 2013; Thacker 2015), and so were our results. *Synechogobius* was embedded in the *Acanthogobius* clade, supporting the previous view that *Synechogobius* may be a synonym of *Acanthogobius* (Birdsong et al. 1988; Pezold 2011). *Lophiogobius* was placed at the basal of this clade, which is partially consistent with the result obtained from morphological analysis (Birdsong et al. 1988). It is surprising that *Favonigobius gymnauchen*, known to be the species of the *Gobiopsis*-lineage in Gobiidae (Agorreta et al. 2013; Thacker 2015), belongs to the *Acanthogobius*-lineage in our results.

Given that the freshwater taxa occupied the base of our tree, it could be assumed that the ancestor of Gobioidei originated from freshwater environments (see also Thacker and Hardman 2005; Alfaro et al. 2018). Since our RASP result showed that ancestral Gobioidei was distributed in West Pacific region, odontobutids probably migrated to the northeast to form the contemporary distribution, across Southeast to East Asian freshwater systems. The divergence of Eleotridae likely occurred around Eocene, and ancestral Eleotridae colonized South Pacific at late Oligocene, and the ancestor of *Eleotris* subsequently colonized the Northwest Pacific during Miocene to Pliocene (see also Ozawa 2009). Ancestral butids were likely distributed in West Pacific when inferred based on our RASP analysis, though this taxon is currently distributed across a wide range of areas encompassing Indian Ocean, West and Northwest Pacific regions (Thacker 2009). Considering the fossil records, the potential distribution of butids might be much wider, from freshwater to marine environments during Miocene (Gaudant 1996). Scientists have hypothesized that Mediterranean butids were exterminated by the migration of gobiid fishes at early Miocene (Gierl et al. 2013), which was partially supported by our data showing that Mediterranean gobiids have existed since Miocene (see also Schwarzhans 2010).

Family-level divergences in Gobioidei predominantly occurred during Eocene. While the divergences among gobiid lineages are concentrated around early Miocene (see also Thacker 2015), Oxudercidae lineages likely occurred rather more recently, mid-Oligocene. It is known that the biodiversity of IMPA (Indo-Malay-Philippines Archipelago) increased during late Miocene to Oligocene with the expanded coral reef area and shallow environments along the complex coastlines, due to the collision between Eurasian and Indian plates (Bellwood and Wainwright 2002; Williams and Duda 2008). The enormous rate of speciation around IMPA could begin with adaptive radiation to various habitats and subsequent spatial isolation. Based on the timing and biogeographic information presented in this study, the immense species diversity of Gobiidae and Oxudercidae at least partially coincides with the explosive speciation in IMPA.

Our estimation of divergence time between Gobiidae and Oxudercidae is congruent with a previous result (Near et al. 2012). Ancestral Oxudercidae likely arose at late Eocene through the West to Northwest Pacific Ocean. The vast majority of Gobiidae species live primarily in marine habitats, indicating that the diversity of Gobiidae might have been shaped under the influence of biological or physical environments of marine habitats (Thacker and Hardman 2005). Some Oxudercidae lineages distributed in Northwest Pacific may have invaded into freshwater habitats from Pliocene to Pleistocene, which might be the beginning of the widespread adaptive radiation. The time of oxudercid freshwater invasion is coincident with the divergence time of freshwater species in other taxa (Goto et al. 2015), indicating that there may have been an environmental situation that allowed migration to freshwater, such as coastline deformation or sea level fluctuation. Our 12S rRNA analysis, at least partially, provided insight into the differentiation and adaptation of Oxudercidae species that were not analyzed in previous studies. Particularly, the evidence for the differentiation and dispersion of the Oxudercidae taxa, which appeared in Miocene and Pleistocene, adapted to the sea or brackish environment, can be considered to be of great importance.

## Data Availability

The data that support the findings of this study are openly available in the NCBI database (https://www.ncbi.nlm.nih.gov/nuccore) under GenBank Accession Numbers KM030423 to KM030442.
